# Gynecologists’ perspectives on two types of uterus-preserving surgical repair of uterine descent; sacrospinous hysteropexy versus modified Manchester

**DOI:** 10.1007/s00192-020-04568-y

**Published:** 2020-10-26

**Authors:** Rosa A. Enklaar, Brigitte A. B. Essers, Leanne ter Horst, Kirsten B. Kluivers, Mirjam Weemhoff

**Affiliations:** 1 Department of Obstetrics and Gynecology, Zuyderland Medical Center, Heerlen, The Netherlands; 2 Department of Obstetrics and Gynecology, Radboud university Nijmegen Medical Center, Geert Groote plein Zuid 10, 6525 GA Nijmegen, The Netherlands; 3 Department of Clinical Epidemiology and Medical Technology Assessment, University Hospital Maastricht, Maastricht, The Netherlands

**Keywords:** Pelvic organ prolapse, Uterine preservation, Hysteropexy, Manchester, Preference

## Abstract

**Introduction and hypothesis:**

The modified Manchester (MM) and sacrospinous hysteropexy (SSH) are the most common uterus-preserving surgical procedures for uterine descent. Little is known about gynecologists’ preferences regarding the two interventions. The study’s aim was to identify which factors influence Dutch (uro)gynecologists when choosing one of these techniques.

**Methods:**

This qualitative study consists of ten semi-structured interviews with Dutch (uro)gynecologists using predetermined, open explorative questions, based on a structured topic list**.** An inductive content analysis was performed using Atlas.ti.

**Results:**

For SSH, the majority (6/10 gynecologists) reported the more dorsal change of direction of the vaginal axis as a disadvantage and expected more cystocele recurrences (7/10). The most reported disadvantage of MM was the risk of cervical stenosis (7/10). Four gynecologists found MM not to be appropriate for patients with higher stage uterine prolapse. The quality of the uterosacral ligaments was related to the chance of recurrence according to five gynecologists. Patient counseling was biased toward one of the uterus-preserving operations (7/10). Four gynecologists stated they make the final decision while two let patient-preference lead the final decision.

**Conclusions:**

Preference for one of the uterus-preserving interventions is mainly based on the gynecologist’s own experience and background. The lack of information regarding these two uterus-preserving procedures hampers evidence-based decision making, which explains the practice pattern variation. In conclusion, further research is needed to improve evidence-based counseling and shared decision making regarding the choice of procedure.

**Electronic supplementary material:**

The online version of this article (10.1007/s00192-020-04568-y) contains supplementary material, which is available to authorized users.

## Introduction

Pelvic organ prolapse (POP) is a common disorder among vaginally parous women. Due to the many different surgical techniques and the lack of a strong evidence-based consensus, there is a large variation in surgical treatment of uterine prolapse worldwide [1–3]. Until recently, vaginal hysterectomy (VH) was the operation of choice. However, the importance of uterus-preserving procedures in the surgical management of POP has been recognized as a result of recent publications where sacrospinous hysteropexy (SSH) showed a non-inferior or even better outcome [4–9]. This has led to a growth in the number of uterus-preserving procedures for POP worldwide [3, 10, 11].

Studies have shown that in addition to scientific evidence, other factors such as patient preferences, surgeon training, and financial and logistic factors influence a surgeon’s choice [12]. Currently, there is no information about how these factors influence gynecologists when choosing one of the specific uterus-preserving techniques. In a 10-year follow-up survey, Jha et al. showed wide practice variation and an increase of 10% in the number of uterus-preserving operations for uterine descent, including SSH and MM [3, 13, 14]. In The Netherlands, the number of POP operations has also increased by 113%. Although more operations were conducted, the number of VH operations decreased by almost 40%. This indicates a trend toward vaginal uterus-preserving surgery [10]. In recent years, of the total prolapse surgeries, there have been an average of 2203 SSH procedures (20%) and 662 MM procedures (5%), according to DIS open data [healthcare declaration information system; a Dutch database provided by the Dutch Healthcare Authority (NZa)].

There is little scientific evidence comparing SSH and MM, thus insufficient information to make evidence-based decisions with respect to the optimal technique. The aim of this study was to identify the considerations of Dutch (uro)gynecologists and influencing factors when choosing a uterus-preserving technique: SSH or MM.

## Materials and methods

### Study design

A qualitative study consisting of semi-structured interviews with Dutch gynecologists was conducted. All interviews were done by one researcher (LtH) and took place either in a face-to-face setting or by telephone or videoconference call. The interview protocol included an introduction, and the gynecologists were asked predetermined, open explorative questions, based on a structured topic list.

The participating gynecologists were representative of the population of Dutch gynecologists with a urogynecology subspecialization or special interest in urogynecology. A selection of gynecologists with different characteristics (gender, age, academic/non-academic, years since completing residency) was made and was furthermore based on their known preferences and (extensive) clinical experience with at least one uterus-preserving technique. In this way getting insights from all perspectives was facilitated.

### Data processing and analysis

The interviews were fully transcribed with the transcribing program f4 GmbH version 3.1.0. The transcripts were analyzed by two researchers (RE and LtH) using the qualitative analysis tool Atlas.ti GmbH version 8.3.20 (Berlin, Germany). Beforehand, a preliminary topic list was made of possible factors contributing to the choice for a specific type of operation technique. During the interviews the topic list was complemented in case an argument was not yet on the existing list. The content was analyzed by inductive content analysis. Open coding was applied, staying semantically close to the original words. The codes with similar content were then divided into themes representing the arguments’ content [15]. Data are presented as numbers and percentages and median and interquartile range as appropriate.

## Results

After eight interviews, saturation of data was reached as no new topics were added to the topic list. To confirm saturation, two additional interviews were conducted, which means that in total ten gynecologists took part. The topics could be categorized in the following themes: patient characteristics, physician’s characteristics and operational or technical aspects. These themes were divided into several subcategories (Appendix 1. Code tree). All gynecologists stated that they were able to speak freely. The interviews took between 20 and 45 min. The interviews were done by telephone (3), by videoconference (1) or in a face to face conversation (6).

The characteristics of the participating gynecologists are shown in Table 1. Advantages and disadvantages of the operation techniques are shown in Table 2. Some of the advantages or disadvantages were mentioned for both procedures, depending on the preference of the gynecologist.

**Table 1:** Characteristics of respondents

Characteristics	n (%), *N* = 10
Sex	
Male	3 (30)
Female	7 (70)
Age (years) median (IQR*)	47 (44.5–48.5)
Practice type	
Academic teaching hospital	2 (20)
Non-academic teaching hospital	8 (80)
Years since completing residency median (IQR*)	13 (9–14)
Preferred procedure	
Sacrospinous hysteropexy (SSH)	6 (60)
Modified Manchester (MM)	4 (40)
Experience procedures per gynecologist per year, median, (IQR*)	
Sacrospinous hysteropexy	45 (21–56)
Modified Manchester	12 (9–30)
Procedure performance	
Both MM and SSH	8 (80)
Only MM	1 (10)
Only SSH	1 (10)

Data are presented as numbers of respondents (%)

*IQR = interquartile range

**Table 2:** Advantages and disadvantages mentioned by gynecologists for SSH and MM

Sacrospinous hysteropexy		Modified Manchester	
Advantages		Advantages	
Ease of procedure	6/10	Ease of procedure	5/10
Firmer fixation	6/10	Normal anatomy kept intact	5/10
Higher elevation	7/10	Suitable in case of elongated cervix	10/10
Shorter operation time	4/10	Lower risk of complications	2/10
Lower risk of complications	1/10		
Less blood loss	1/10		
Disadvantages		Disadvantages	
Change of vaginal axis	6/10	More blood loss	5/10
Dyspareunia	4/10	Dyspareunia	4/10
Higher costs (device)	5/10	Cervical stenosis	7/10
Higher cystocele recurrence rate	7/10	Not suitable for all patients	
Complexity of complications (harder to reach)	4/10	Premenopausal	6/10
Risk of exposure of non-absorbable sutures	3/10	Severe prolapse	4/10
		Less firm fixation	4/10
		Longer operation time	4/10
		Quality of uterosacral ligaments	5/10

### Arguments regarding patient characteristics

According to the gynecologists, the stage of prolapse had an impact on their preference for MM. In case of severe cervical elongation, all gynecologists preferred MM to SSH. However, four gynecologists stated that they preferred SSH to MM in case of a higher stage prolapse. Moreover, the quality of the uterosacral ligaments influenced the choice for MM in five respondents. In cases where the uterosacral ligaments were more stretched, a higher risk of recurrence was expected by five gynecologists. In patients with a high prolapse stage, the quality of the fixation of the uterosacral ligaments in MM is expected to be weaker, and SSH is the procedure of choice by four gynecologists. Menopausal status was mentioned as an important factor for choice of technique by seven respondents; six gynecologists specifically mentioned preferring SSH to MM for premenopausal women because of the cervical amputation and the risk of cervical stenosis after MM.

### Arguments regarding physician’s characteristics

The majority (6/10) of the gynecologists mentioned that they would be willing to learn a new technique or improve their skills. The other four gynecologists felt secure enough about both operation techniques. Six gynecologists mentioned that they learned one or both operation techniques during their residency. Their experiences in combination with the experiences of their older or more experienced colleagues in their team were leading in the general preferences and techniques used in the hospitals.

### Arguments regarding operational and technical aspects

Both SSH and MM require identical capacity such as scrub nurse assistance during the operation. SSH was perceived as being more expensive than MM when applying the closed approach, as this requires a device. The operation time and costs were not regarded as factors with an impact on the decision-making process.

The gynecologists differed in their views regarding the anatomical results of the procedures. Seven mentioned the high elevation of the uterus in SSH as a positive anatomical result. In contrast, two considered the apical prolapse correction in SSH to be non-physiologically high. The risk of recurrence of cystocele after SSH was mentioned by seven respondents. Although the apical fixation of the uterus in SSH was described as a firm fixation by six gynecologists, three different gynecologists had the same opinion on regarding MM. Four gynecologists stated that dyspareunia after SSH was expected to be caused by the firm fixation to the sacrospinous ligament and therefore less mobility of the uterus in comparison with MM. However, those who expected more dyspareunia to occur after MM mentioned the uterosacral sutures and more sensitive cervix post-amputation as a cause of dyspareunia.

In younger premenopausal patients, higher complication rates were mentioned in respect to MM: more perioperative blood loss caused by a more vascularized cervix, risk of developing hematometra or menstrual problems such as dysmenorrhea caused by cervical stenosis. Cervical stenosis caused by the cervical amputation was considered a disadvantage by seven gynecologists, thereby making intra-uterine device placement or endometrial ablation more complex in case indicated at a later stage. In both procedures a higher risk of de novo dyspareunia was mentioned in premenopausal women because a higher sexual activity was expected in younger patients. Some gynecologists preferred SSH as the operation of choice in older women with (multiple) comorbidities as they considered it to be less invasive: a simpler procedure (6/10 gynecologists) with a short operation time (4/10 gynecologists).

### Counseling

Seven gynecologists reported that their counseling was biased toward one of the two uterus-preserving operations, mostly based on their own experiences and preference in that specific case. Only two stated that the patient’s wish was leading in their decision. Three gynecologists said that they make the final decision based on their findings after examination and then counseled toward one operation. One gynecologist makes the final decision but does explain the different techniques to the patient. Furthermore, two of the gynecologists make the decision themselves, because they were convinced discussing the hysterectomy and the two uterus-preserving techniques would either overwhelm the patient or would be too much complex information for the patients to understand and oversee the consequences.

## Discussion

In this qualitative study we have explored the different perspectives of Dutch gynecologists with an urogynecology subspecialization or special interest in urogynecology concerning surgical choices about surgical uterus-preserving treatment of pelvic organ prolapse.

Maher et al. described in their Cochrane review that the choice for a specific prolapse repair, among others, depends on the type and severity of the prolapse and the general health of the women [8]. This was in line with the preferences in our interviews [4, 7].

In our study, recurrence of anterior compartment prolapse is one of the most often mentioned disadvantages of the SSH procedure. Data related to the recurrence of cystocele after SSH vary from 11.8% to 47% [4, 16, 17]. Gynecologists noted that the unilateral fixation and high fixation of SSH causes a distortion of the vaginal axis; a stretched, immobile vaginal wall will get different pressure profiles and stretch from the continuous abdominal pressure. Cervical stenosis was regarded as a disadvantage of the MM procedure, as it may cause hematometra or dysmenorrhea and hamper uterine access for, e.g., endometrial sampling. However, literature is scarce on this subject, and the reported percentages of postoperative cervical stenosis differ between 0.7% and 11.3% [18, 19]. Dyspareunia was mentioned as a common disadvantage after SSH and MM procedures. We postulate that all prolapse repairs carry that risk; however, no comparative data are available on this subject. Several studies have shown an improvement of 43% (SSH) and 52% (MM) in sexual functioning after POP with native tissue repair [18, 20–22]. De novo postoperative dyspareunia was reported in 5.6% after MM [18]. There are few data available concerning de novo dyspareunia after SSH; the only data available is regarding vaginal vault prolapse correction, which is often caused by the sacrospinous sutures. As shown in Table 1, there has been more experience with SSH compared with MM among the interviewed gynecologists. This is in agreement with the percentages of both operations conducted in The Netherlands (SSH 20% and MM 5% of the total POP operations in The Netherlands). Less experience might lead to a longer operation time. However, the costs of both techniques were estimated to be equal as the longer operation time of the MM would compensate the higher material costs of SSH. Therefore, these factors were not regarded as factors with an impact on the decision-making process as mentioned earlier.

This study identified the arguments on which the current decision-making process is based. The interviews have shown that these arguments cannot be considered independently, but that a combination substantiates the gynecologist’s preference. Although these influencing factors have not previously been studied, specifically in case of SSH and MM, the professional’s training and experience have been indicated as important influencing factors in practice pattern variation [12]. Detollenaere et al. showed advantages of the uterus-preserving techniques, such as less blood loss and shorter operation, hospital stay and recovery times [4]. Frick et al. showed that when patients were counseled for hysterectomy and uterus-preserving surgery with similar success rates, 60% would prefer uterus-preserving surgery over hysterectomy [23]. This is consistent with the findings of van IJsselmuiden et al. who demonstrated that women with prolapse complaints have a preference for uterus preservation in case equal outcomes after both interventions are expected. After counseling, the preference for uterus-preserving surgery increased by 22% [24]. The patients considered the doctor’s opinion, surgical risk and the risk of developing uterine cancer as leading factors in the decision-making process.

In the interviews it became clear that the counseling for type of surgery is not structured and often limited to what gynecologists consider to be the most necessary parts. An argument for this strategy is that they do not want to overload the patients with information. However, little is known about what aspects of the operations patients consider to be important factors when making a decision. In 2018, a Dutch multicenter RCT was set up to compare the two uterus-preserving techniques: SSH versus MM (SAM study), as well as a study on patients’ preferences (SAM DCE study) [25]. The results of these two trials will contribute to better evidence-based counseling and to more shared decision making, and may reduce practice pattern variation after implementation of the results.

Worldwide, the most common procedure of choice for uterine descent remains the VH [1, 3]. One of the strengths of this study is that Dutch (uro)gynecologists have good insight in the pros and cons of different uterus-preserving procedures in view of the rising popularity of these operations over the last years. This extensive experience ensures that the considerations mentioned in this study are substantiated. A limitation of this study is that the interviewed gynecologists are all from the same country, whereas it would be interesting to see if the perspectives of the Dutch gynecologists toward these operational techniques are shared by their international colleagues. Another limitation is that the interviews were conducted using different methods (by telephone, video conference or face to face). This might cause a slight difference in interaction during the interviews, and in an interview by telephone non-verbal communication is lacking. However, the format of the interviews was standardized to minimize variances, and the majority were conducted face to face or via videoconference. The study furthermore provides an overview on the arguments for doctor’s preferences, but not regarding the frequency of these arguments.

In conclusion, we have explored the different perspectives of Dutch gynecologists concerning surgical choices related to surgical uterus-preserving treatment of POP. The preference for one of the uterus-preserving interventions was mainly based on the gynecologist’s own experiences and background. Patient-related factors, training, experience and opinions of key colleagues or team members were important factors regarding a gynecologist’s preference for SSH or MM. Differences in technical or organizational aspects were not found to be arguments that favored either intervention. A striking finding is the high variability in the way the gynecologists counseled the patients, e.g., shared decision making or counseling directed toward one of the two uterus-preserving operations. The current lack of evidence on the comparison of these two uterus-preserving procedures hampers evidence-based decisions and allows practice pattern variation to arise.

## Electronic supplementary material

ESM 1 (DOCX 15 kb)
