# Supplementary material for: Gynecologists’ perspectives on two types of uterus-preserving surgical repair of uterine descent; sacrospinous hysteropexy versus modified Manchester
Source: Int Urogynecol J. 2020 Oct 26;32(4):835–40. doi: 10.1007/s00192-020-04568-y (PMC8009770; doi:10.1007/s00192-020-04568-y)
Supplement: Supplementary file 1 — (DOCX 15 kb) [file 192_2020_4568_MOESM1_ESM.docx]

## Appendix 1. Code tree

| **Code tree** |  |  |
| --- | --- | --- |
| **Patient characteristics** | **Physician's characteristics** | **Operation/technical** |
| **Anatomy** | **Education** | **Organizational** |
| Quality of ligaments | Education during residency/obgyn training | Costs |
| Location of ligaments | Education after residency/obgyn training | Material |
| Elongatio collis | Learning curve | Complexity of operation |
| Stage of prolapse | **Experience** | Duration of operation |
| **Health status** | Expertise | **Perioperative problems** |
| Abnormality of uterus | Willing to improve skills | Complication risk |
| Comorbidity | Opinion | Perioperative blood loss |
| Impact of operation | Distrust in operation | **Result of operation** |
| Age of patient | **External influences** | Effect on vaginal walls |
| Premenstrual status | Necessary assistance | Quality of fixation |
| **Patients’ choice** | Expertise operation team | Postoperative anatomical result |
| Preference patient | Image of operation | Level of elevation of uterus |
| Counseling | Forgotten | Percentage of recurrence |
|  | Opinion of colleague(s) | **Postoperative problems** |
|  | Residents in training | Cervical stenosis |
|  | Research | Defecation problems |
|  |  | Duration of recovery |
|  |  | Micturition complaints |
|  |  | Postoperative pain |
|  |  | Reaction to non-absorbable sutures |
|  |  | Sexual complaints |
|  |  | Recurrence of cystocele |
